# Propionic Acidemia: An Ambispective Cohort Study on Phenotypic and Genotypic Characteristics at a Tertiary Center in Vietnam

**DOI:** 10.3390/genes17091099

**Published:** 2026-09-11

**Authors:** Diep Minh Quang, Chi Dung Vu, Khanh Ngoc Nguyen, Ngoc Lan Nguyen, Tran Thi Chi Mai, Nguyen Thi Thanh Huong, Do Thi Mo, Le Thi Phuong, Thinh Huy Tran, Nguyen Huu Tu, Ngo Xuan Khoa, Vu Viet Ha Vuong, Van Khanh Tran

**Affiliations:** 1 Hanoi Medical University, 1st Ton That Tung Street, Hanoi 11520, Vietnam; dr.diepminhquang1989@gmail.com (D.M.Q.); dungvu@nch.gov.vn (C.D.V.); khanhnn@nch.gov.vn (K.N.N.); tranchimai@hmu.edu.vn (T.T.C.M.); nguyenhuutu@hmu.edu.vn (N.H.T.); ngoxuankhoavn@gmail.com (N.X.K.); 2 Haiphong International Hospital of Obstetrics and Pediatrics, 124 Nguyen Duc Canh Street, Haiphong 04721, Vietnam; 3 Center of Endocrinology, Metabolism, Genetic/Genomics and Molecular Therapy, Vietnam National Children’s Hospital, 18/879 La Thanh, Hanoi 11528, Vietnam; domo@nch.gov.vn; 4 Center for Gene and Protein Research, Hanoi Medical University, 1st Ton That Tung Street, Hanoi 11520, Vietnam; nguyenngoclan@hmu.edu.vn (N.L.N.); phuongle@hmu.edu.vn (L.T.P.); 5 Department of Biochemistry, Vietnam National Children’s Hospital, 18/879 La Thanh, Hanoi 11528, Vietnam; 6 Department of Diagnostic Imaging, Vietnam National Children’s Hospital, 18/879 La Thanh, Hanoi 11528, Vietnam; thanhhuong.nguyenrad82@gmail.com; 7 Biochemistry Department, Hanoi Medical University, 1st Ton That Tung Street, Hanoi 11520, Vietnam; tranhuythinh@hmu.edu.vn; 8 Hospital of Post and Telecommunications, Phuong Liet, Hanoi 11412, Vietnam; hapro2609@gmail.com

**Keywords:** propionic acidemia, Vietnamese, *PCCB*, prenatal diagnosis

## Abstract

**Background/objective**: Propionic acidemia (PA), an autosomal recessive metabolic disorder, is caused by biallelic pathogenic variants in the *PCCA* or *PCCB* genes. In Vietnam, molecular analyses of PA have been scarce. Hence, the objective of this study was to investigate the phenotypic and genotypic characteristics and clinical management of Vietnamese children with PA. **Methods**: An ambispective descriptive cohort study was conducted on 14 children who were diagnosed with PA at the Vietnam National Children’s Hospital. Collected data encompassed clinical manifestations, biochemical profiles, imaging characteristics, genetic findings, clinical management, and outcomes. **Results**: Symptom onset within the cohort spanned the neonatal, early infantile, and early childhood periods, with a median age at onset of 7.50 months (range 0.33–34.00 months). The clinical phenotype was characterized by acute encephalopathy, accompanied by lethargy (64.3%), vomiting (57%), and seizures (43%). Even though all tested cases exhibited abnormal urinary organic acids and elevated propionylcarnitine (C3) concentration, other key laboratory findings comprised hyperammonemia (100%), metabolic acidosis (64%), and hematological alterations (64%). The study identified eight pathogenic or likely pathogenic *PCCB* variants and two variants of uncertain significance, with the c.1124C>T being the most frequently identified variant. This cohort had severe clinical outcomes, as neurodevelopmental impairment was observed in 79% of cases with a mortality rate of 43%. **Conclusions**: This study underscores the severe clinical and biochemical characteristics of PA in 14 Vietnamese children, reinforcing the necessity of early detection and comprehensive long-term care for PA patients. Furthermore, the identification of 10 *PCCB* variants, including the recurrent c.1124C>T, p.(Ala375Val) variant in our cohort, broadens the known *PCCB* variant spectrum.

## 1. Introduction

Propionic acidemia (PA, OMIM #606054) is a congenital metabolic disorder inherited as an autosomal recessive trait first identified in 1961 by Childs et al. [1]. It is marked by a deficiency of the enzyme propionyl-CoA carboxylase (PCC; EC 6.4.1.3), a biotin-dependent mitochondrial enzyme catalyzing the conversion of propionyl-CoA to methylmalonyl-CoA, which then enters the Krebs cycle via succinyl-CoA [2]. This step is pivotal in the catabolism of branched-chain amino acids (isoleucine, valine, methionine, and threonine), odd-chain fatty acids, and cholesterol side chains [2,3]. The absence of PCC activity induces the accumulation of propionic acid and related metabolites, including propionylcarnitine (C3), 2-methylcitrate (MCA), propionylglycine, and 3-hydroxypropionate (3HPA) [4,5,6].

Regarding clinical presentation, PA manifests marked phenotypic heterogeneity, ranging from severe neonatal-onset to milder late-onset forms [3,7], with the neonatal-onset form being the most prevalent. The severe neonatal-onset form typically presents within the first months of life, with symptoms including poor feeding, vomiting, lethargy, hypotonia, seizures, and tachypnea because of metabolic acidosis; these symptoms can rapidly progress to coma and death. The milder late-onset form usually manifests after several months or years. It is marked by recurrent episodes of metabolic decompensation associated with infection, fasting, or stress, with symptoms, including neurological dysfunction, hypotonia, recurrent vomiting, hyperammonemia, and acidosis [7,8,9,10].

PA is a rare genetic metabolic disorder, with its incidence varying with geographic location, from 0.05–5.05 per 100,000 (Asia-Pacific) to 3.6–8.14 per 100,000 (Middle East and North Africa) [9], with higher prevalence documented in Greenland (1 in 1000) and with an estimated average ranging from 1 in 50,000 [11] to 1 in 100,000 live births worldwide [12].

Propionyl-CoA carboxylase is a 750 kDa heterododecameric enzyme comprising six α-subunits and six β-subunits (α6β6), encoded by the *PCCA* (OMIM *232000) and *PCCB* (OMIM *232050) genes, respectively [2]. The *PCCA* gene, located on chromosome 13q32.3, comprises 24 exons and encodes the α-subunit of propionyl-CoA carboxylase, whose primary function is biotin carboxylation, a process playing a pivotal role in maintaining overall enzyme activity—a critical step in the enzymatic reaction. The *PCCB* gene, located on chromosome 3q22.3, comprises 15 exons and encodes the β-subunit of propionyl-CoA carboxylase, which contains the catalytic carboxyltransferase domain essential for enzymatic function. The β-subunit is integral to substrate recognition and catalysis, and its proper assembly with the α-subunit is required for forming a functional PCC holoenzyme [13,14].

PA is caused by pathogenic variants in *PCCA* and *PCCB*, engendering partial or complete dysfunction of the PCC enzyme. To date, a wide spectrum of *PCCA* and *PCCB* variants has been documented, with each gene harboring over 100 variants, including missense, nonsense, splice-site, and small insertions/deletions [15]. Missense mutations are the most prevalent variant type and often affect highly conserved residues that are involved in enzyme stability or catalytic activity [16,17]. Even though genotype–phenotype correlations in PA remain complex, patients with *PCCB* mutations are typically reported to have a relatively milder clinical course than those with *PCCA* mutations [15,18]. However, severe neonatal-onset disease has also been documented in *PCCB*-associated PA [8,15,19].

PA treatment focuses on controlling and preventing the long-term accumulation of propionic acid and related metabolites by using L-carnitine and metronidazole, restricting propionate-producing amino acids, limiting micronutrient supplementation, and regular monitoring [20,21,22]. Nevertheless, PA remains associated with substantial morbidity and impaired quality of life despite advances in newborn screening and metabolic management [22,23]. Molecular genetic analysis of the *PCCA* and *PCCB* genes is pivotal for confirming the diagnosis of PA and for facilitating carrier detection, prenatal diagnosis, and genetic counseling [24,25].

To date, no studies in Vietnam have employed next-generation sequencing to identify pathogenic variants in individuals with PA. Therefore, the objective of this study was to delineate the genotype and phenotype spectra, as well as the clinical management, of Vietnamese children with confirmed PA.

## 2. Materials and Methods

### 2.1. Study Design and Participants

This ambispective descriptive cohort study examined children with a confirmed diagnosis of propionic acidemia at the Vietnam National Children’s Hospital. The inclusion criteria were (1) clinical manifestations indicating inborn errors of metabolism, such as vomiting, poor feeding, lethargy, seizures, coma, and unexplained metabolic acidosis; (2) biochemical markers, such as elevated C3 levels and abnormal urinary organic acid profiles; and (3) the presence of disease-causing variants in *PCCA* or *PCCB*. Exclusion criteria comprised incomplete clinical, biochemical, or molecular data.

Research conducted at a tertiary-care center may overrepresent patients with severe clinical presentations. Therefore, to enhance case identification, we cross-checked the hospital database with records from the regional newborn screening network. The tandem mass spectrometry (MS/MS)-based newborn screening program for inborn errors of metabolism is currently being implemented on a pilot basis in Northern Vietnam and encompasses approximately 3–13% of newborns. During the period represented in the newborn screening database, 526,494 newborns in the region received MS/MS screening. We included all patients with confirmed propionic acidemia who met the study eligibility criteria.

### 2.2. Data Collection

Following the Institutional Review Board approval on 30 June 2023, data collection was undertaken until December 2025. This process encompassed the retrospective extraction of historical medical records for patients diagnosed before this date, as well as the prospective follow-up of enrolled children. The collected dataset comprised the following: (1) clinical parameters: age at onset, age at diagnosis, clinical course, neurological status, treatment response, and clinical outcome; and (2) biochemical profiles: blood pH, bicarbonate, blood ammonia, homocysteine, liver enzymes, blood glucose, quantitative acylcarnitine analysis with the principal marker C3 and the C3-to-acetylcarnitine (C2) ratio (C3/C2), and urinary organic acid analysis; and (3) genetic findings.

### 2.3. Genetic Testing

Genomic DNA was isolated from peripheral blood with the QIAamp DNA Blood Kit (Qiagen, Hilden, Germany) using the manufacturer’s instructions. Exome sequencing was conducted at the Center for Gene-Protein Research, Hanoi Medical University using the MGI system (BGI, Beijing, China). Panel sequencing was undertaken by Invitae (Invitae Corporation, San Francisco, CA, USA). The average depth of coverage for Invitae multigene panels was approximately 300×–500×, with targeted regions sequenced to at least 50×. Reads were aligned to the GRCh37 reference genome. The assay attained >99% analytical sensitivity and specificity for single-nucleotide variants, insertions and deletions < 15 bp, and exon-level deletions and duplications. A comprehensive organic acidemia panel used for sequencing and filtering of variants comprised *ACAD8* (NM_014384.2), *ACADSB* (NM_001609.3), *ACAT1* (NM_000019.3), *ACSF3* (NM_174917.4), *ASPA* (NM_000049.2), *AUH* (NM_001698.2), *BCKDHA* (NM_000709.3), *BCKDHB* (NM_183050.2), *BTD* (NM_000060.3), *D2HGDH* (NM_152783.4), *DBT* (NM_001918.3), *DHTKD1* (NM_018706.6), *DLD* (NM_000108.4), *DNAJC19* (NM_145261.3), *ETFA* (NM_000126.3), *ETFB* (NM_001985.2), *ETFDH* (NM_004453.3), *ETHE1* (NM_014297.3), *FBP1* (NM_000507.3), *FH* (NM_000143.3), *FTCD* (NM_006657.2), *GCDH* (NM_000159.3), *GSS* (NM_000178.2), *HIBCH* (NM_014362.3), *HLCS* (NM_000411.6), *HMGCL* (NM_000191.2), *HSD17B10* (NM_004493.2), *IDH2* (NM_002168.3), *IVD* (NM_002225.3), *L2HGDH* (NM_024884.2), *MCCC1* (NM_020166.4), *MCCC2* (NM_022132.4), *MCEE* (NM_032601.3), *MLYCD* (NM_012213.2), *MMAA* (NM_172250.2), *MMAB* (NM_052845.3), *MMACHC* (NM_015506.2), *MMADHC* (NM_015702.2), *MUT* (NM_000255.3), *NFU1* (NM_001002755.2), *OGDH* (NM_002541.3), *OPA3* (NM_025136.3), *OPLAH* (NM_017570.4), *OXCT1* (NM_000436.3), *PCCA* (NM_000282.3), *PCCB* (NM_000532.4), *POLG* (NM_002693.2), *PPM1K* (NM_152542.4), *SERAC1* (NM_032861.3), *SLC13A5* (NM_177550.4), *SLC25A1* (NM_005984.4), *SLC25A19* (NM_021734.4), *SUCLA2* (NM_003850.2), *SUCLG1* (NM_003849.3), *TAZ* (NM_000116.4), and *TMEM70* (NM_017866.5).

The pathogenicity of the identified variants was predicted using the Combined Annotation Dependent Depletion (CADD v1.7) [26] and Rare Exome Variant Ensemble Learner (REVEL v1.3) tools [27]. For variant classification, the guidelines of the American College of Medical Genetics and Genomics (ACMG) and the Association for Molecular Pathology (AMP) were utilized [28], incorporating refinements from ClinGen Variant Classification Guidance (https://www.clinicalgenome.org/tools/clingen-variant-classification-guidance/; accessed on 14 July 2026). REVEL scores were interpreted using ClinGen SVI-calibrated thresholds for computational evidence [29]. The reference transcript used for *PCCB* was NM_000532.4.

### 2.4. Management and Outcome Assessment

To manage acute metabolic decompensation and long-term care, we followed the Guidelines for the Diagnosis and Management of Methylmalonic Acidemia and Propionic Acidemia [22].

Regarding long-term outcomes, neurodevelopmental status was evaluated using standardized developmental and psychological tools, neurological examinations, and behavioral evaluations. For children under 5 years of age, developmental screening was performed using the Denver II test [30]. For children over 5 years of age, non-verbal intelligence was assessed using Raven’s Progressive Matrices [31]. Attention-deficit/hyperactivity disorder (ADHD) and related behavioral symptoms were specifically evaluated using the Vanderbilt ADHD Diagnostic Rating Scale [32]. In accordance with the updated International Classification of Diseases, 11th Revision (ICD-11) framework, patients were classified as having neurodevelopmental impairment if they exhibited disorders of intellectual development (developmental quotient [DQ] or intelligence quotient [IQ] < 70), specific neurodevelopmental or neurobehavioral disorders (e.g., attention-deficit/hyperactivity disorder [ADHD] with developmental speech delay), or isolated functional motor impairments. A patient presenting with borderline intellectual functioning (e.g., an IQ of 80) without overt functional impairments was categorized as borderline and placed under close clinical surveillance.

### 2.5. Prenatal Testing

All parents, verified as obligate carriers, received genetic counseling regarding the autosomal recessive inheritance pattern and the 25% recurrence risk in subsequent pregnancies. During the study, the mother of patient P5 was pregnant; she consented to prenatal testing. Amniocentesis was conducted at 17 weeks of gestation. The amniotic fluid sample was centrifuged at 3000× *g* for 10 min; the supernatant was discarded, and 200 µL of the remaining fluid, along with the pellet, was transferred to a new 1.5 mL Eppendorf tube for DNA extraction. Genomic DNA was extracted from the amniotic fluid using the QIAamp DNA Mini Kit (Qiagen, Hilden, Germany). PCR was conducted, and Sanger sequencing was performed using an ABI 3500 Genetic Analyzer (Applied Biosystems, Foster City, CA, USA). To visualize Sanger sequencing chromatograms, we used SnapGene Viewer version 8.2.2 (Dotmatics, Boston, MA, USA).

### 2.6. Data Analysis

Statistical analyses were performed using SPSS 20.0 (IBM Corp., Armonk, NY, USA). Continuous variables were presented as median with min–max range or mean ± standard deviation (SD) depending on data distribution. Categorical variables were provided as frequencies and percentages. Patients were stratified into three onset groups: neonatal onset (within the first 4 weeks of life; *n* = 3), early infantile onset (one to six months of age; *n* = 4), and late-onset (greater than 6 months of age; *n* = 7). This stratification was conducted to examine whether clinical and biochemical features, as well as management strategies, differed across these subgroups. To compare groups, we used the Chi-square test or Fisher’s exact test, as appropriate. To compare continuous variables, we used One-Way ANOVA or the Kruskal–Wallis test, depending on the data distribution. When a significant result was obtained for a non-normally distributed variable, the Mann–Whitney U test was used for post-hoc pairwise comparisons. A *p*-value < 0.05 was considered significant. However, the reported *p*-values were descriptive; no adjustment for multiple comparisons was applied, and nominally significant results should be interpreted as signals to be confirmed in larger cohorts. A Kaplan–Meier analysis, with age as the time scale, was conducted to account for delayed entry (left truncation). The entry time was set as the age at disease onset. Even though death was considered the event, patients who remained alive were censored at their age at last follow-up. This delayed-entry approach accounts for differences in age at disease onset and precludes treating patients as being at risk before the onset of clinically recognized disease. Median overall survival age and corresponding 95% confidence intervals (CIs) were determined. Kaplan–Meier analyses were conducted using RStudio version 2025.09.0+387.

## 3. Results

### 3.1. Demographic, Clinical, and Biochemical Characteristics

This study involved a total of 14 children from 14 unrelated, non-consanguineous Vietnamese families diagnosed with PA. Table 1 and Supplementary Table S1 illustrate the demographic, clinical, and biochemical characteristics. The median age at onset was 7.50 months (range: 0.33–34.00 months) (Table 1). The most common symptoms encompassed lethargy (64%), vomiting (57%), seizures, poor feeding, and hypotonia (43% each) (Table 1). Tachypnea was observed in 5/14 cases (36%), skin lesions in 3/14 cases (21%), and hypothermia in one case (7.1%). Biochemical analysis confirmed severe metabolic disturbances, with 14/14 cases showing abnormal urinary organic acids and 12/12 cases demonstrating elevated C3 levels (Table 1). Hyperammonemia (14/14; 100%), metabolic acidosis (9/14; 64%), hematological alterations (9/14; 64%), and elevated anion gap (8/14; 57%) were predominant. Among patients with metabolic acidosis, 8 of 9 (89%) presented with an elevated anion gap (Supplementary Table S1). The hematological profile comprised anemia (50.0%), leukopenia (43%), and thrombocytopenia (21%). Elevated glycine was detected in 4/10 patients, and elevated liver enzymes in 3/14 patients. No cases of hypoglycemia were observed.

The overall median age at diagnosis was 9.00 months (range: 0.53–34.20 months). Among three onset groups, most clinical features and biochemical parameters were similar. Hypotonia was more frequent with earlier disease onset (*p* = 0.044), affecting 100% (3/3) of neonatal-onset cases, 50% (2/4) of early infantile-onset cases, and 14% (1/7) of late-onset cases. Differences in NH_3_ concentrations were detected across the three onset groups (H = 6.013, *p* = 0.036). Post-hoc pairwise comparisons using the Mann–Whitney U test revealed that NH_3_ levels were higher in the neonatal onset group (Mean rank = 11.33) than the late-onset group (Mean rank = 4.86) (*p* < 0.05). Due to the small sample size, these subgroup comparisons should be interpreted with caution.

### 3.2. Genetic Testing Results

**Figure 1:**
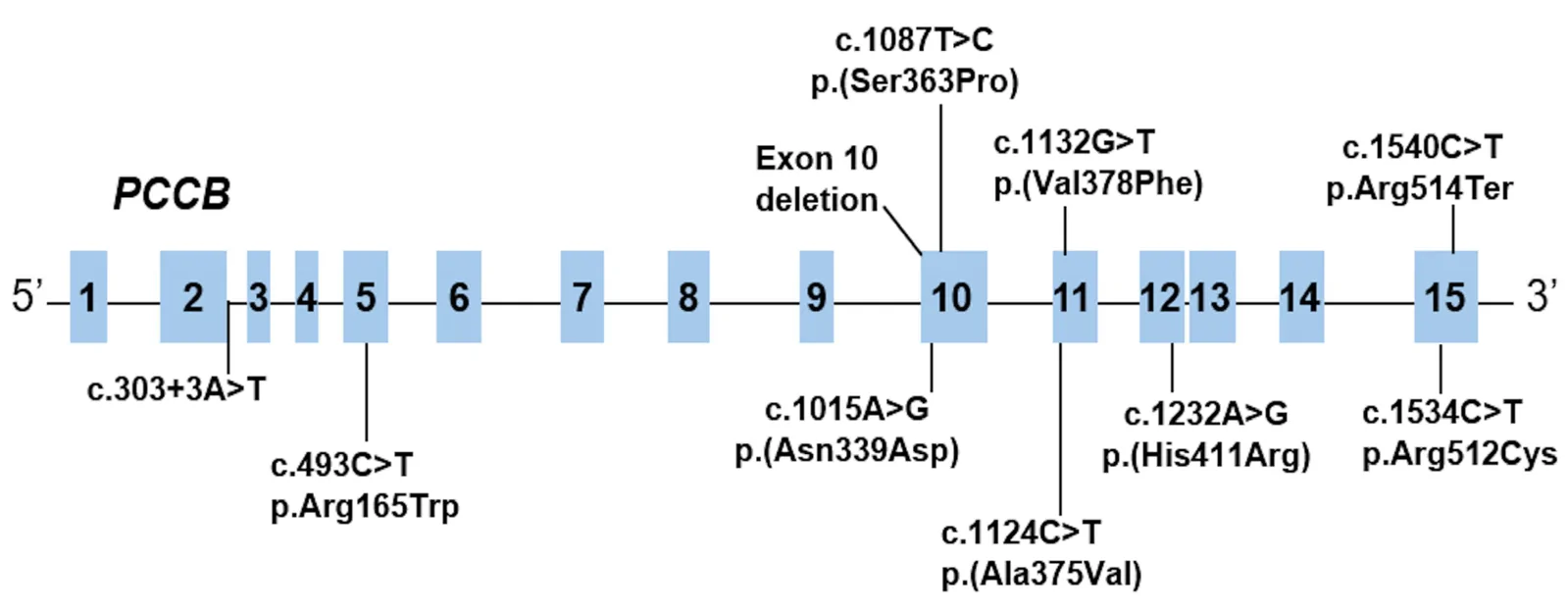
Locations of *PCCB* variants identified in 14 Vietnamese children with propionic acidemia.

Table 2 and Figure 1 illustrate that a total of 10 different *PCCB* variants were identified in 14 children, located in intron 2 and exons 5, 10, 11, 12, and 15. The most frequently observed variant was c.1124C>T, p.(Ala375Val), accounting for 13 of 28 alleles (46.4%). Other recurrent variants comprised c.303+3A>T; c.1087T>C, p.(Ser363Pro); and c.1132G>T, p.(Val378Phe). Six unique variants were identified: c.493C>T, p.Arg165Trp; c.1015A>G, p.(Asn339Asp); exon 10 deletion; c.1232A>G, p.(His411Arg); c.1534C>T, p.Arg512Cys; and c.1540C>T, p.Arg514Ter. Eight variants were classified as pathogenic or likely pathogenic, and two as variants of uncertain significance according to ACMG guidelines (Table 2 and Supplementary Table S2). Of these, four variants have been previously documented in PA patients, while six variants have not been reported in the literature. All 14 children harbored the variants in either homozygous or compound heterozygous states.

### 3.3. Management and Clinical Outcomes

Acute clinical management involved restriction of protein intake, administration of high-energy support with intravenous glucose (with or without lipids), and supplementation with L-carnitine (100%), vitamin B12, and biotin before definitive diagnosis for all patients (Table 3). Sodium benzoate was administered only to patients with hyperammonemia. After PA was confirmed by urinary organic acid analysis and/or molecular testing, vitamin B12 and biotin were discontinued, whereas L-carnitine supplementation was sustained. While 86% required antibiotics, 71% needed mechanical ventilation, 29% required continuous veno-venous hemofiltration, and 29% required antiepileptic treatment. After 9 to 33 days of treatment, all patients were stabilized and discharged (Supplementary Table S1). No differences were detected in acute management between the neonatal-onset, early infantile-onset, and late-onset groups.

**Figure 2:**
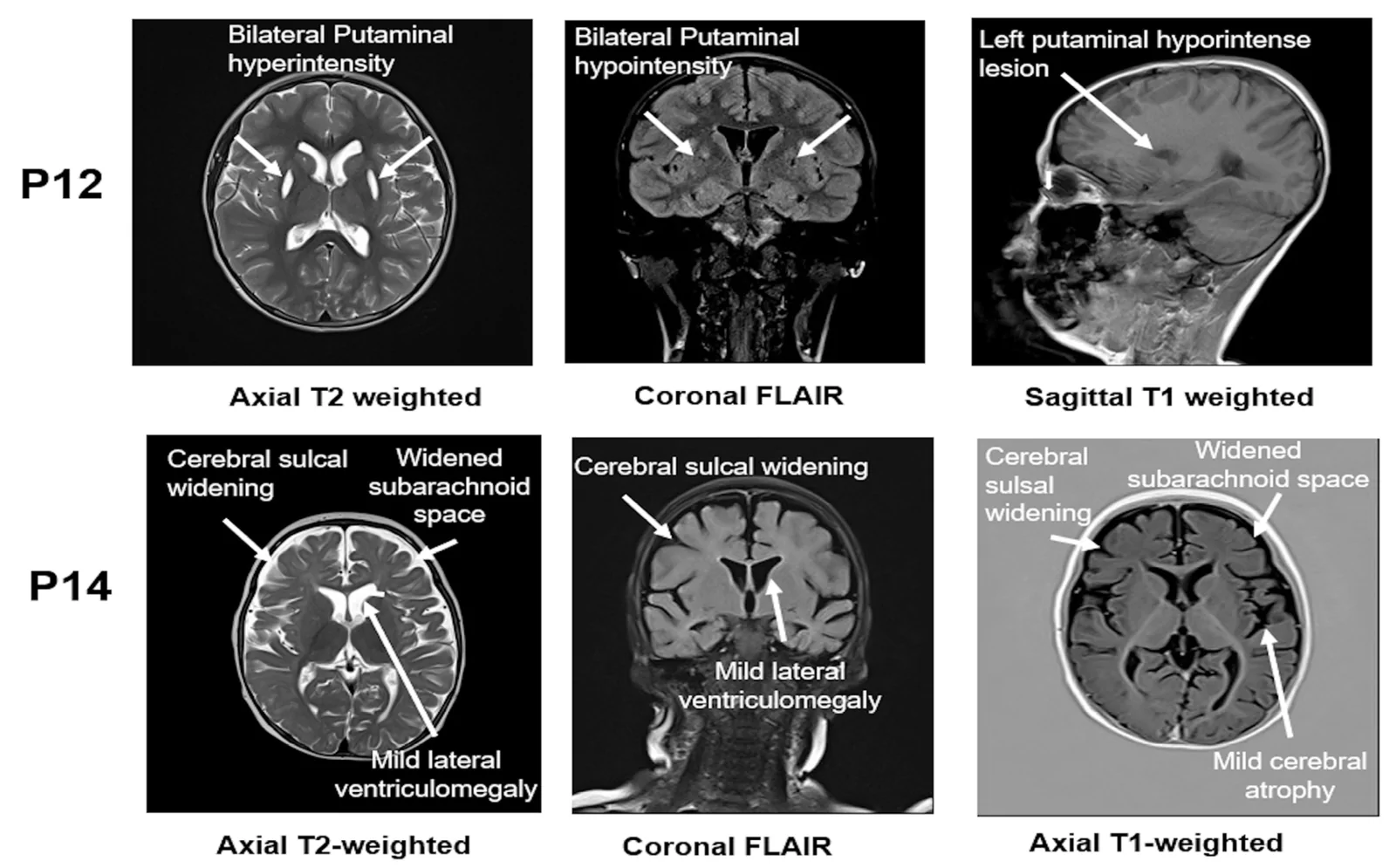
Magnetic resonance imaging of the brain in patients P12 and P14. P12: symmetric hyperintense lesions in bilateral putamen on an axial T2-weighted image; symmetric hypointense lesions in the bilateral putamen with a hyperintense rim on a coronal fluid-attenuated inversion recovery (FLAIR) image; and a hypointense lesion in the left putamen in a sagittal T1-weighted image. P14: widening of the cerebral sulci and subarachnoid space in the supratentorium, associated with mild dilatation of the lateral ventricles on an axial T2-weighted image; widening of the cerebral sulci and the subarachnoid space in the supratentorium, associated with mild dilatation of the lateral ventricles on a coronal FLAIR image; and widening of the cerebral sulci and subarachnoid space in the supratentorium, associated with mild dilatation of the lateral ventricles and consistent with mild cerebral atrophy on an axial T1-weighted image.

All patients received a long-term protein-restricted diet supplemented with specialized formulas, avoidance of fasting, and adequate caloric intake. Regular follow-up was critical to monitoring metabolic control, growth, and complications, including renal and cardiac involvement. Despite this management, neurodevelopmental impairment was detected in 10 of 14 patients (71%), while one patient with a borderline intelligence quotient score was categorized as having borderline intellectual functioning and placed under close clinical surveillance (Table 4). Notably, electroencephalograms demonstrated epileptiform discharges. Neuroimaging revealed numerous structural brain abnormalities, such as basal ganglia involvement, white matter abnormalities, and atrophy. For instance, patient P12 exhibited bilateral cystic degeneration of the lentiform nuclei on magnetic resonance imaging (MRI) (Figure 2). In contrast, patient P14 showed widening of the cerebral sulci and the subarachnoid space in the supratentorial region, along with diffuse cerebral parenchymal atrophy (Figure 2). Cardiac abnormalities were detected in three patients. Patient P6 manifested moderately reduced left ventricular systolic function, mild tricuspid regurgitation, and mild mitral regurgitation. Patient P7 had severely reduced left ventricular systolic function with an ejection fraction of 38%. Patient P10 presented with a small ductus arteriosus. No significant differences were evident in clinical outcomes, survival rates, or sequelae among the neonatal, early infantile, and late-onset groups.

**Figure 3:**
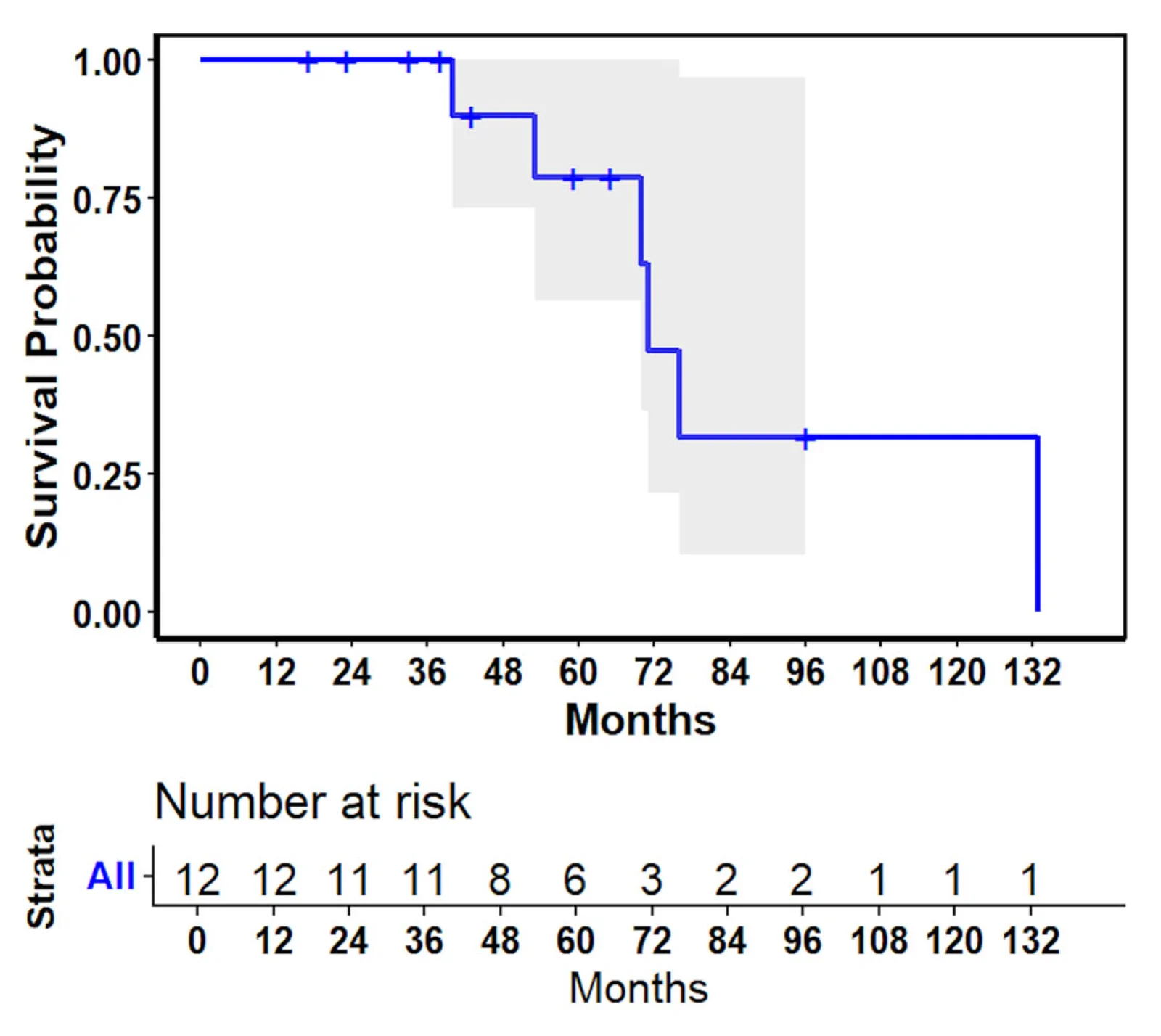
Left-truncated Kaplan–Meier estimate of event-free survival in 14 patients with propionic acidemia. Survival probability is plotted against chronological age (months), with delayed entry incorporated to account for left truncation. The shaded area represents the 95% confidence interval. The risk table below displays the number of patients at risk at each 12-month interval.

During the follow-up period, six deaths were recorded (Table 4). The estimated median overall survival age was 71 months (95% CI: 70–NA). The Kaplan–Meier estimated overall survival probability was 100% (95% CI: 100–100%) at 36 months, which subsequently decreased to 90% (95% CI: 73–100%) at 48 months, and to 79 (95% CI: 56–100%) at 60 months (Figure 3). At older ages, the estimated survival probability continued to decrease, but the confidence intervals became wider, with only a few patients remaining at risk. Accordingly, these later estimates, particularly after 60 months, should be interpreted with caution and may not reliably reflect the long-term natural history of propionic acidemia in this cohort.

All six deaths occurred during episodes of acute metabolic decompensation (Table 4). The deaths were two patients in the neonatal onset group (P13 and P14), two in the early infantile onset group (P4 and P11), and two in the late-onset group (P6 and P10). The ages at death for P4, P13, P14, P10, P6, and P11 were 40, 53, 70, 71, 76, and 133 months, respectively. Whereas sepsis or septic shock accompanied the fatal crisis in three patients (P4, P10, and P13), cardiac involvement with heart failure was reported in two patients (P4 and P6). During the final crisis, marked hyperammonaemia was observed in five of the six patients, with peak NH_3_ concentrations ranging from 126.3 to 438.0 µmol/L in P6, P10, P11, P14, and a lower peak concentration of 88.6 µmol/L in P13. Lactate levels exceeded 16 mmol/L in four patients (P6, P10, P11, and P14), and glucose levels exceeded 19 mmol/L in five patients. Markedly elevated aminotransferase levels were detected in P13 (aspartate aminotransferase, 883.4 U/L; alanine aminotransferase, 466.2 U/L) and P6 (aspartate aminotransferase, 163.6 U/L). Moreover, P6 had a creatine kinase level of 6937 U/L, a troponin I level of 0.622 µg/L, and an N-terminal pro-B-type natriuretic peptide level of 2159 pg/mL; all of these findings were consistent with severe cardiac injury.

### 3.4. Prenatal Testing Findings in a Family

**Figure 4:**
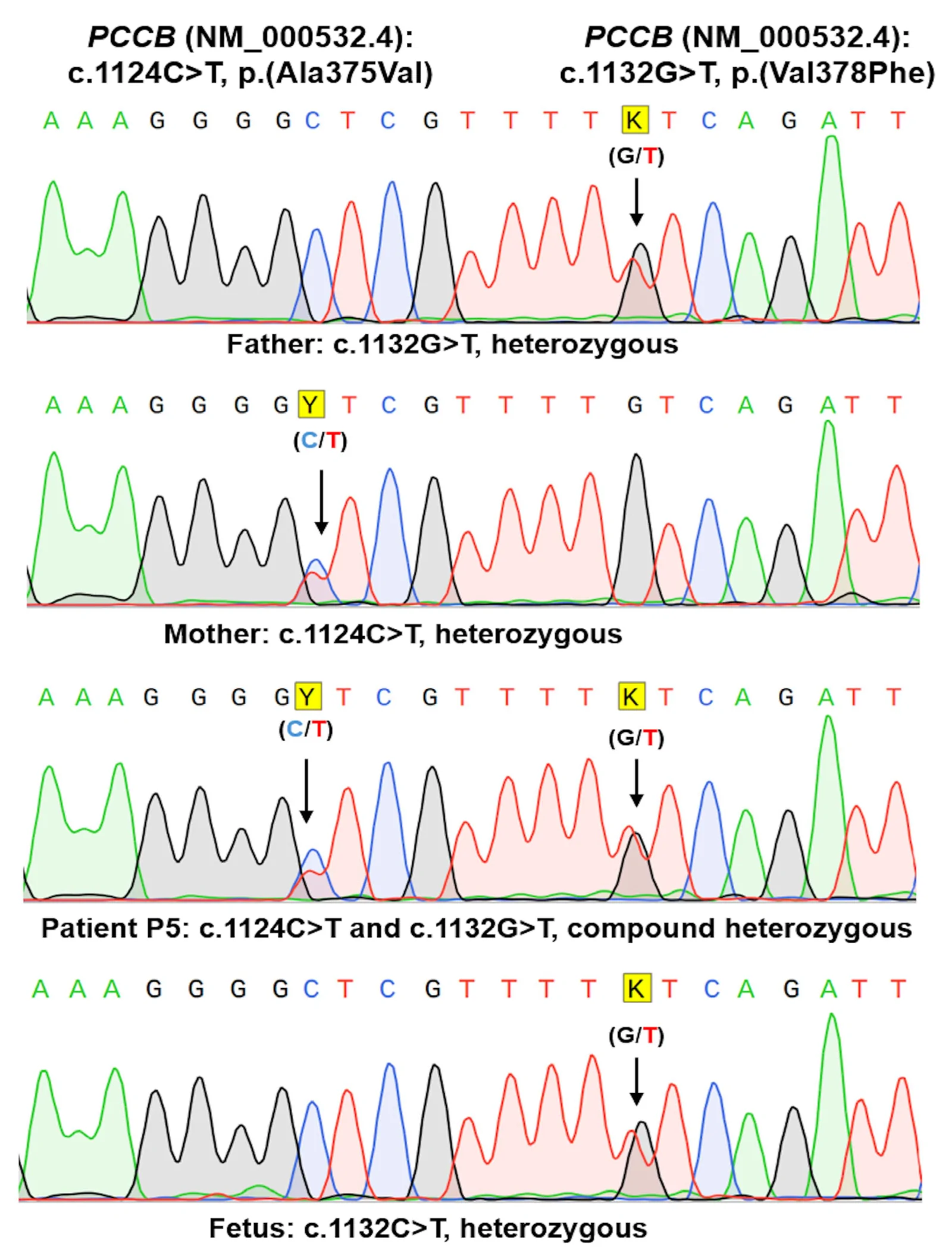
Electropherogram of Sanger sequencing of *PCCB*: c.1124C>T, p.(Ala375Val) and c.1132G>T, p.(Val378Phe) in the family of patient P5. The father harbored c.1132G>T, p.(Val378Phe) in the heterozygous state. The mother carried c.1124C>T, p.(Ala375Val) in the heterozygous state. The proband P5 harbored a compound heterozygote. The fetus carried c.1132G>T, p.(Val378Phe) in the heterozygous state. Heterozygous nucleotide positions are indicated by arrows and highlighted using International Union of Pure and Applied Chemistry (IUPAC) ambiguity codes: Y = C/T and K = G/T.

During the study, the mother of patient P5 was pregnant and consented to prenatal testing. The father harbored c.1132G>T (p.(Val378Phe)) in a heterozygous state (Figure 4). The mother carried c.1124C>T (p.(Ala375Val)) in a heterozygous state (Figure 4). However, the fetus inherited only the c.1132G>T (p.(Val378Phe)) variant in a heterozygous state from the father, and the pregnancy continued (Figure 4). Postnatal metabolic screening revealed no abnormalities, confirming an unaffected phenotype.

## 4. Discussion

In this study, the main clinical symptoms comprised vomiting, neurological impairment, and coma, accompanied by severe biochemical abnormalities, including elevated C3 and C3/C2 ratios, hyperammonemia, and metabolic acidosis. In the study, 10 *PCCB* variants (including eight pathogenic or likely pathogenic variants and two variants of uncertain significance) were identified, with c.1124C>T recurrently observed in this cohort. Even though acute episodes can be managed according to established protocols, long-term management has been challenging, with a mortality rate of up to 43%.

Our patients demonstrated typical biochemical features of PA, including elevated C3 levels, elevated C3/C2 ratios, and elevated levels of propionic acid, 3-hydroxypropionic acid, and methylcitrate. Other increased compounds were 3-hydroxyvaleric acid, 2-methyl-3-hydroxybutyric acid, 2-methyl-3-hydroxyvaleric acid, 4-hydroxyphenylpyruvic acid, propionylglycine, tiglylglycine, lactic acid, 3-hydroxybutyric acid, aceton acetic acid, and 3-hydroxyvaline. These findings align with those reported by Liu et al. (2022) [15] and are more frequent than in the studies by Kör et al. (2019) [33] and Kim et al. (2002) [34].

In this study, we observed that ammonia levels and hypotonia tended to be associated with earlier onset. Higher ammonia concentrations were observed in patients with neonatal-onset (270 µmol/L) than in those with the late-onset group (105 µmol/L). Consistent with established mechanisms, this secondary hyperammonemia may promote the neurological burden, driving the high incidence of hypotonia and other complications in the neonatal cohort. Aligning with this metabolic severity, hypotonia was more prevalent in the neonatal group (100%) than in only 14% of late-onset cases. The small sample size (*n* = 14) in the study may make these comparisons exploratory; thus, they should be interpreted with caution.

In this study, we identified several previously reported *PCCB* variants: c.493C>T, p.Arg165Trp; c.1087T>C, p.(Ser363Pro); c.1534C>T, p.Arg512Cys; and c.1540C>T, p.Arg514Ter. The variant c.493C>T, p.Arg165Trp has previously been associated with a relatively milder or late-onset phenotype when present in compound heterozygosity, with residual PCC activity [35,36,37]. In our cohort, patient P8 had late-onset disease at 34 months and harbored the compound heterozygous variants, c.303+3A>T and c.493C>T, presenting with vomiting, gait ataxia, and a normal brain MRI. The c.1087T>C, p.(Ser363Pro) was observed in two patients with late-onset disease. Although its functional effect has not been directly demonstrated, a different substitution at the same residue, p.Ser363Leu, has been reported in a patient with severe PA, indicating that this residue may be important for protein function [38,39]. The variant c.1534C>T, p.Arg512Cys has been associated with profound protein instability and impaired enzyme assembly, and has been linked to a severe neonatal-onset phenotype [36,37]. In our cohort, one female patient was homozygous for this variant and showed a severe clinical phenotype, including reduced left ventricular systolic function (ejection fraction 38%) and a developmental quotient of 45%. These findings indicate that the homozygous c.1534C>T, p.Arg512Cys genotype is associated with a severe PA phenotype. The nonsense variant c.1540C>T, p.Arg514Ter has previously been associated with loss of enzymatic activity, neurological impairment, and early mortality [18,40,41]. In our cohort, patient P6 harbored the compound heterozygous variants c.1087T>C and c.1540C>T, had late-onset disease with cardiac involvement, including moderately reduced left ventricular systolic function, tricuspid regurgitation, and mild mitral regurgitation. He died at the age of 6.4 years.

Six additional variants were identified in our cohort: c.303+3A>T, c.1015A>G, c.1124C>T, c.1132G>T, c.1232A>G, and exon 10 deletion. Notably, the variant c.1124C>T, p.(Ala375Val) was a recurrent variant in our cohort. Several common *PCCB* variants have been documented in different ethnic populations, including c.1301C>T, p.Ala434Val in Chinese patients [42]; c.1304T>C, p.Tyr435Cys in Japanese patients [19]; c.425G>A, p.Gly142Asp in Saudi Arabian patients [39]; and c.1218_1231delinsTAGAGCACAGGA, p.Gly407Argfs14 in Caucasian populations [14]. Our findings broaden the known spectrum of *PCCB* variants. The recurrent c.1124C>T, p.(Ala375Val) and c.1132G>T, p.(Val378Phe) within the carboxyltransferase (CT) domain may suggest regional enrichment within this Vietnamese population. To our understanding, our study is the first to report c.303+3A>T, a splice-site variant predicted to have a high SpliceAI score of 0.90, in a patient with PA. The missense variants c.1015A>G and c.1232A>G may affect protein function, while the exon 10 deletion further highlights the molecular heterogeneity of *PCCB* deficiency in Vietnamese patients with PA.

Propionic acidemia (PA) demonstrates a broad spectrum of clinical outcomes, ranging from early mortality to long-term neurodevelopmental impairment. Neurodevelopmental impairment is highly prevalent in patients with PA, affecting 71% of our cohort, which is consistent with reported findings of 60–90% [15,43,44]. Mortality is high, particularly in the severe neonatal-onset cases, ranging from 15% to 46% globally [15,23,45]. In our cohort, the mortality rate was 43%, higher than in several published cohorts but comparable to the findings of Akar et al. (2025) [45]. The deaths in our cohort occurred during acute metabolic crises, frequently accompanied by infection, hyperammonaemia, or cardiac complications, rather than during periods of clinical stability. The occurrence of deaths across different onset groups posits that the severity of acute metabolic crises may be a critical determinant of outcome irrespective of age at onset. The high mortality rate should also be interpreted with caution, as our hospital is a tertiary referral center and hence likely receives a greater proportion of severely affected patients.

Kaplan–Meier analysis revealed no deaths before age 3 in this cohort. The estimated median overall survival age was 71 months, with decreased estimated survival probabilities at later ages. Nevertheless, these later estimates should be interpreted with caution due to the small sample size and the limited number of patients remaining at risk. Long-term complications, including cardiomyopathy, neurological deterioration, recurrent metabolic crises, and severe infections, may be associated with mortality in this period [46]. Accordingly, early detection and comprehensive long-term care are required for patients with PA. Newborn screening is an effective approach for early detection and enhanced outcomes in PA [15,43]. However, in our cohort, only one patient (P9) was identified through newborn screening. This circumstance may be largely related to the limited coverage of newborn screening in Vietnam and may not accurately demonstrate the true prevalence of PA in the population. Consequently, milder, asymptomatic, or late-onset cases may remain undetected, generating potential ascertainment bias in our cohort.

In our study, prenatal diagnosis was successfully conducted for the family of patient P5. Genetic testing indicated that the fetus harbored the c.1132G>T, p.(Val378Phe) variant in the heterozygous state. This experience emphasizes the clinical utility of genetic testing to confirm the diagnosis in affected patients and to support accurate prenatal testing in subsequent pregnancies. Prenatal diagnosis for known pathogenic variants is routinely implemented in PA [7,24,25,47] and, when combined with biochemical analysis, improves diagnostic accuracy, especially for families with incomplete genetic findings [24]. Moreover, preimplantation genetic testing provides a proactive reproductive option for couples affected by PA, enabling the transfer of an unaffected embryo [47,48,49]. Reinforcing genetic counseling services and enhancing access to prenatal diagnosis and preimplantation genetic testing may substantially benefit affected families.

This study has several limitations. First, as a single-center study, our sample size is small, indicating the rarity of PA. Consequently, the precision of the survival estimates and the ability to establish genotype–phenotype correlations remained limited. Nonetheless, as the leading national tertiary referral hospital in Vietnam, our institution maintains one of the largest and most clinically diverse PA cohorts in the country. Additionally, our genotypic and phenotypic findings may serve as a valuable reference for molecular diagnostics and clinical management in Vietnam and neighboring regional populations. Second, the ambispective design and the tertiary referral setting in our study may have introduced selection, information, referral, and ascertainment biases. Patients with more severe presentations would be more likely to be referred to our hospital. In contrast, milder, asymptomatic, or late-onset cases might have been missed, especially because newborn screening coverage in the region remained limited. For the prognostic analysis, we implemented a Kaplan–Meier approach with age as the timescale to account for delayed entry (left truncation) and decrease survivorship bias. These potential sources of bias should be considered when interpreting the long-term outcomes and the generalizability of our findings. Moreover, prenatal testing in our study was restricted to a single family with a previously affected child. Nevertheless, this successful clinical intervention reveals the technical feasibility of high-risk molecular diagnosis and the critical role of genetic counseling for patients with PA and other inherited metabolic disorders in Vietnam. Finally, even though several previously unreported variants were identified in our study, their pathogenicity was inferred using silico prediction and clinical evidence. Additional in vitro functional studies are required to elucidate their biological effects and confirm their pathogenic role. However, the detailed documentation of these variants in our cohort may serve as a valuable reference for future regional genomic research and clinical variant interpretation.

## Figures and Tables

**Table 1:** Demographic, clinical and biochemical characteristics of 14 patients with propionic acidemia at first evaluation.

Characteristics	Value	Neonatal Onset	Early Infantile Onset	Late-Onset	*p* Value
Number of patients	14 (100%)	3 (21%)	4 (29%)	7 (50%)	
Age at onset, median (Min–Max) (months)	7.50 (0.33–34.00)	0.48 (0.33–0.63)	3.75 (2.50–5.00)	13.00 (7.50–34.00)	0.000233 *
Age at diagnosis, median (Min–Max) (months)	9.0 (0.53–34.2)	0.65 (0.53–0.77)	5.5 (2.5–27.0)	13.0 (7.5–34.2)	0.015 *
Gender (*n* = 14)					0.133 **
Male (%)	9 (64%)	3 (100%)	1 (25%)	5 (71%)	
Female (%)	5 (36%)	0 (0%)	3 (75%)	2 (29%)	
Clinical symptoms (*n* = 14)					
Lethargy	9 (64%)	3 (100%)	2 (50%)	4 (57%)	0.476 **
Vomiting	8 (57%)	0 (0%)	3 (75%)	5 (71%)	0.149 **
Hypotonia	6 (43%)	3 (100%)	2 (50%)	1 (14%)	0.044 **
Seizure	6 (43%)	1 (33%)	2 (50%)	3 (43%)	1.000 **
Poor feeding	6 (43%)	3 (100%)	1 (25%)	2 (29%)	0.149 **
Tachypnea	5 (36%)	1 (33%)	2 (50%)	2 (29%)	0.790 **
Skin lesion	3 (21%)	1 (33%)	1 (25%)	1 (14%)	1.000 **
Hypothermia	1 (7.1%)	0 (0%)	0 (0%)	1 (14%)	1.000 **
Biochemical characteristics					
C3 (*n* = 12), mean ± SD (µmol/L)	10.98 ± 4.00	8.98 ± 2.20 (*n* = 2)	13.91 ± 3.13 (*n* = 4)	9.69 ± 4.22 (*n* = 6)	0.204 ***
Elevated C3 (*n* = 12)	12 (100%)	2 (100%)	4 (100%)	6 (100%)	
NH_3_ (*n* = 14), median (Min–Max) (µmol/L)	122 (65–655)	270 (134–655)	162.5 (93–284)	105 (65–154)	0.036 *
NH_3_ mean rank ^#^		11.33 ^a^	9.25 ^ab^	4.86 ^b^	
Hyperammonemia (*n* = 14)	14 (100%)	3 (100%)	4 (100%)	7 (100%)	
Metabolic acidosis (*n* = 14)	9 (64.3%)	2 (67%)	2 (50%)	5 (71%)	0.790 **
Anion gap (*n* = 14), mean ± SD (mmol/L)	16.19 ± 6.01	13.97 ± 6.20	13.98 ± 7.37	18.41 ± 5.17	0.417
Elevated anion gap (*n* = 14)	8 (57%)	2 (67%)	1 (25%)	5 (71%)	
Abnormal urinary organic acids (*n* = 14)	14 (100%)	3 (100%)	4 (100%)	7 (100%)	
Glycine (*n* = 10), median (Min–Max) (µmol/L)	396.00 (204–1814)	1100 (*n* = 1)	340 (204–738) (*n* = 3)	396 (225–1814) (*n* = 6)	0.502 *
Hyperglycinemia (*n* = 10)	4 (40%)	1 (100%)	1 (33%)	2 (33%)	0.714 **
Leukopenia (*n* = 14)	6 (43%)	1 (33%)	3 (75%)	2 (29%)	0.385 **
Anemia (*n* = 14)	7 (50%)	0 (0%)	3 (75%)	4 (57%)	0.241 **
Thrombocytopenia (*n* = 14)	3 (21%)	1 (33%)	0 (0%)	2 (29%)	0.538 **
Elevated liver enzymes (*n* = 14)	3 (21%)	1 (33. %)	1 (25%)	1 (14%)	1.000 **

C3, propionylcarnitine; NH_3_, ammonia; *n*, number of patients. * Kruskal–Wallis test; **, Fisher’s Exact test; ***, One-way ANOVA. ^#^ Values with different superscript letters (a, b) indicate differences in NH_3_ concentrations between pairs of groups based on the Mann–Whitney U post-hoc test, conducted without Bonferroni correction for exploratory purposes.

**Table 2:** *PCCB* variants identified in 14 patients with propionic acidemia in this study.

c.DNA Change	Amino Acid Change	Patient (Status of Variant)	REVEL Score	CADD	Previously Reported in PA	dbSNP	ClinVar Status	ACMG Classification
c.303+3A>T	p.?	P3 (hom) P8 (het)	-	D	Not reported	-	4521649	VUS (PM3_Moderate, PM2_Supporting, PP3, PP4)
c.493C>T	p.Arg165Trp	P8 (het)	0.930	D	Reported	rs879253815	P (217894)	Pathogenic (PS3, PM1, PM3_Moderate, PP3_Moderate, PM2_Supporting, PP4)
c.1015A>G	p.(Asn339Asp)	P12 (het)	0.945	D	Not reported	rs1934921365	VUS (939293)	VUS (PP3_Moderate, PM2_Supporting, PP4)
c.1087T>C	p.(Ser363Pro)	P1, P6 (het)	0.945	D	Reported	rs770341883	P (939354)	Likely Pathogenic (PM3_Strong, PP3_Moderate, PM2_Supporting, PP4)
Exon 10 deletion	-	P10 (het)	-	-	Not reported	-	-	Pathogenic (PVS1, PM3_Moderate, PM2_Supporting, PP4)
c.1124C>T	p.(Ala375Val)	P1, P2, P5, P11, P12 (het) P4, P9, P13, P14 (hom)	0.972	D	Not reported	rs1576360099	VUS (939294)	Likely Pathogenic (PM3_Strong, PP3_Moderate, PM2_Supporting, PP4)
c.1132G>T	p.(Val378Phe)	P5, P10, P11 (het)	0.977	D	Not reported	rs761529521	VUS (859270)	Likely Pathogenic (PM3_Strong, PP3_Moderate, PM2_Supporting, PP4)
c.1232A>G	p.(His411Arg)	P2 (het)	0.925	D	Not reported	-	VUS (4059323)	Likely Pathogenic (PM3_Moderate, PP3_Moderate, PM2_Supporting, PP4)
c.1534C>T	p.Arg512Cys	P7 (hom)	0.921	D	Reported	rs186710233	P (38879)	Pathogenic (PS3, PM3_Strong, PP3_Moderate, PM2_Supporting, PP4)
c.1540C>T	p.Arg514Ter	P6 (het)	-	D	Reported	rs749908889	P (217893)	Pathogenic (PVS1_Strong, PS3, PM3_Strong, PM2_Supporting, PP4)

ACMG, American College of Medical Genetics and Genomics; PA, propionic acidemia; REVEL, Rare Exome Variant Ensemble Learner; CADD, Combined Annotation Dependent Depletion; dbSNP, Database of Single Nucleotide Polymorphisms; VUS, variant of uncertain significance; P, pathogenic; LP, likely pathogenic; hom, homozygous; het, heterozygous; D, damaging; PS, strong pathogenic evidence; PM, moderate pathogenic evidence; PP, supporting pathogenic evidence; -, not available; p.?, unknown protein-level consequence.

**Table 3:** Acute management of 14 patients with propionic acidemia.

	Total (*n* = 14)	Onset Stage			
		Neonatal Onset (*n* = 3)	Early Infantile Onset (*n* = 4)	Late-Onset (*n* = 7)	*p* Value (Fisher’s Exact Test)
Protein restriction	14/14 (100%)	3/3 (100%)	4/4 (100%)	7/7 (100%)	
IV Glucose infusion	14/14 (100%)	3/3 (100%)	4/4 (100%)	7/7 (100%)	
L-carnitine (IV) ^c^	14/14 (100%)	3/3 (100%)	4/4 (100%)	7/7 (100%)	
Hydroxocobalamin (Vitamin B12) ^a^	14/14 (100%)	3/3 (100%)	4/4 (100%)	7/7 (100%)	
Biotin ^a^	14/14 (100%)	3/3 (100%)	4/4 (100%)	7/7 (100%)	
Sodium benzoate ^b^	14/14 (100%)	3/3 (100%)	4/4 (100%)	7/7 (100%)	
Antibiotics	12/14 (86%)	3/3 (100%)	3/4 (75%)	6/7 (86%)	1.000
Continuous veno-venous hemofiltration (CVVH)	4/14 (29%)	2/3 (67%)	1/4 (25%)	1/7 (14%)	0.252
Antiepileptics	4/14 (29%)	1/3 (33%)	2/4 (50%)	1/7 (14%)	0.608
Mechanical ventilation	10/14 (71%)	2/3 (67%)	4/4 (100%)	4/7 (57%)	0.357

IV, intravenous; CVVH, continuous veno-venous hemofiltration; Vitamin B12, hydroxocobalamin; *n*, number of patients. ^a^ Administered empirically before diagnostic confirmation and discontinued once propionic acidemia was confirmed. ^b^ Administered only in patients with hyperammonemia. ^c^ Continued after diagnostic confirmation as part of ongoing management.

**Table 4:** Outcomes of 14 patients with propionic acidemia.

	Brain MRI	Electrophysiology (EEG)	Neurodevelopmental Assessment	Age at Last Follow-Up (Months)	Metabolic Crises (*n*)	Outcome (Cause of Death)
Group 1: Neonatal onset						
P9	Brain atrophy (at 31 mo)	Right hemisphere background slowing (at 17 mo).	Disorders of intellectual development (Reduced DQ: 32 at 23 mo) ^a^	33	10	Alive
P13	Not performed	Epileptiform discharges (at 20 mo)	Global developmental delay ^a^	53	15	Deceased (Septic shock, sepsis, and multiorgan failure during acute metabolic decompensation)
P14	Diffuse cerebral atrophy (at 9 mo)	Not performed	Global developmental delay ^a^	70	31	Deceased (Multiorgan failure during acute metabolic decompensation)
Group 2: Early infantile onset						
P3	White matter lesions (at 4 mo)	Epileptiform discharges (at 4 mo)	Disorders of intellectual development (Reduced DQ: 60) ^a^	43	11	Alive
P4	Brain atrophy; corpus callosum lesion (at 4 mo)	Abnormal EEG (at 4 mo)	Normal development	40	11	Deceased (Septic shock, heart failure, and acute metabolic decompensation)
P7	Not performed	Not performed	Disorders of intellectual development (Reduced DQ: 45 at 17 mo) ^a^	17	2	Alive
P11	Symmetric T2 hyperintensity bilateral caudate and putamina (at 132 mo)	Not performed	Normal development (at 132 mo)	133	2	Deceased (Respiratory and circulatory failure during acute metabolic decompensation)
Group 3: Late onset						
P1	Not performed	Not performed	Normal development	38	6	Alive
P2	Not performed	Not performed	Isolated gross motor impairment (DQ: 70 at 23 mo) ^a^	23	8	Alive
P5	Bilateral putaminal T2 hyperintensity (at 15 mo)	Epilepsy (at 16 mo).	Disorders of intellectual development (at 65 mo) ^a^	65	14	Alive
P6	Not performed	Not performed	ADHD with developmental speech delay (at 52 mo) ^a^	76	13	Deceased (Heart failure and acute metabolic decompensation)
P8	Normal (at 35 mo).	Not performed	Disorders of intellectual development (Reduced DQ: 45 at 45 mo) ^a^	59	5	Alive
P10	Bilateral putaminal lesions (at 32 mo)	Not performed	Global developmental delay (at 32 mo) ^a^	71	5	Deceased (Decompensated PA with septic shock and multiorgan failure)
P12	Bilateral cystic lentiform nuclei (at 93 mo).	Not performed	Borderline intellectual functioning (IQ 80 at 93 mo) ^b^	96	7	Alive

MRI, magnetic resonance imaging; EEG, electroencephalogram; DQ, developmental quotient; IQ, intelligence quotient; ADHD, attention-deficit/hyperactivity disorder; mo, months. ^a^ Patients are included in the neurodevelopmental impairment group (total of 10/14 patients). ^b^ Patient with borderline intellectual functioning (IQ = 80) without overt functional impairments, categorized separately and placed under ongoing developmental surveillance.

## Data Availability

The original contributions presented in this study are included in the article/Supplementary Materials. Further inquiries can be directed to the corresponding author.

## Supplementary Materials

The following supporting information can be downloaded at https://www.mdpi.com/article/10.3390/genes17091099/s1, Table S1: Demographic, clinical, biochemical characteristics, and treatment of 14 patients with propionic acidemia at first admission. Table S2: Classification of 10 *PCCB* variants according to recommendations of the American College of Medical Genetics and Genomics (ACMG) [50,51,52,53].
