# Supplementary material for: Propionic Acidemia: An Ambispective Cohort Study on Phenotypic and Genotypic Characteristics at a Tertiary Center in Vietnam
Source: Genes (Basel). 2026 Sep 11;17(9):1099. doi: 10.3390/genes17091099 (PMC13606846; doi:10.3390/genes17091099)
Supplement: Supplementary file 1 [file genes-17-01099-s001.zip › genes-4479847-supplementary.pdf]

Supplementary Materials

Supplementary Table S1. Demographic, clinical, biochemical characteristics, and treatment of 14 patients with propionic acidemia at first admission

No.	Sex	Age of onset	CLINICAL FEATURES (at admission)	LABORATORY FINDINGS (at admission)											TREATMENT (at admission)	
				C3 (µmol/L)	C3/C2	Urinary organic acids	NH_3 (µmol/L)	Glycine (µmol/L)	Metabolic acidosis	Anion gap (mmol/L)	Hb (g/L)	Platelets ($\times 10^9$/L)	WBC ($\times 10^9$/L)	AST/ALT (U/L)	Treatment approach	Initial outcome
				Ref range: 0.21–4.74	Ref ragne: <0.25	PA-specific markers	Ref range: < 100 if age ≤ 28 days<br>< 50 if age > 28 days	Ref range: 81-436		Ref range: 7-16						
**P1**	M	14.0 mo	Vomiting; Lethargy; Tachypnea; Hypothermia; Skin lesion	5.23 (Elevated)	0.25 (Elevated)	3-HP; 3-OH-Val; MCA elevated	65 (Elevated)	N/A	+	21.8 (Elevated)	123 (Normal)	293 (Normal)	8.17 (Normal)	25/15 (Normal)	Mechanical ventilation; Sodium benzoate; Protein restriction;<br>IV glucose infusion;<br>Antibiotics;<br>IV L-carnitine; Arginine;<br>Hydroxocobalamin (vitamin B12);<br>Biotin;<br>Dietary management.	Stabilized; discharged after 15 days
**P2**	M	7.5 mo	Lethargy; Seizure; Tac hypnea; Poor feeding	17.00 (Elevated)	0.58 (Elevated)	3-HP; MCA; PG elevated	68 (Elevated)	392 (Normal)	+	19.8 (Elevated)	110 (Normal)	328 (Normal)	10.36 (Normal)	48.5/22.3 (Normal)	Oxygen supplementation;<br>Sodium benzoate; Protein restriction;<br>IV glucose infusion;<br>Antibiotics;<br>IV L-carnitine;<br>Hydroxocobalamin (vitamin B12);<br>Biotin;<br>Dietary management.	Stabilized; discharged after 19 days
**P3**	F	4.0 mo	Vomiting; Hypotonia; Lethargy; Seizure; Tachypnea	10.10 (Elevated)	1.74 (Elevated)	PA elevated	141 (Elevated)	340 (Normal)	+	12.9	97 (Anemia)	219 (Normal)	1.33 (Leukopenia)	35/21 (Normal)	Mechanical ventilation; Sodium benzoate; Protein restriction;<br>IV glucose infusion;<br>Antibiotics;<br>IV L-carnitine;<br>Hydroxocobalamin (vitamin B12);<br>Biotin;<br>Antiepileptics (Keppra; Sabril);<br>Dietary management.	Stabilized; discharged after 14 days
**P4**	M	3.5 mo	Seizure	14.35 (Elevated)	1.93 (Elevated)	PA elevated	284 (Elevated)	738 (Elevated)	−	8.9	98 (Anemia)	397 (Normal)	1.39 (Leukopenia)	47.8/37.5 (Normal)	Mechanical ventilation; Protein restriction;<br>IV glucose infusion;<br>IV L-carnitine;<br>Sodium benzoate;<br>Hydroxocobalamin (vitamin B12);<br>Biotin; isoleucine;<br>Antiepileptics (Depakin);<br>Dietary management.	Stabilized; discharged after 14 days.
**P5**	F	9.0 mo	Lethargy; Seizure	8.71 (Elevated)	0.26 (Elevated)	3-HP; MCA; PG elevated	110 (Elevated)	225 (Normal)	+	27.2 (Elevated)	101 (Anemia)	239 (Normal)	5.32 (Normal)	25/16 (Normal)	Mechanical ventilation; Protein restriction;	Stabilized; discharged

															IV glucose infusion;<br>CVVH;<br>Antibiotics;<br>IV L-carnitine;<br>Sodium benzoate;<br>Hydroxocobalamin (vitamin B12);<br>Biotin;<br>Antiepileptics (Keppra);<br>Dietary management.	after 17 days.
**P6**	M	30.0 mo	Vomiting; Poor feeding	6.08 (Elevated)	1.02 (Elevated)	3-HP elevated	106 (Elevated)	1814 (Elevated)	−	12.3	104 (Anemia)	455 (Normal)	0.92 (Leukopenia)	48/41 (Normal)	Protein restriction;<br>IV glucose infusion; sodium benzoate, abatuni,<br>IV L-carnitine;<br>Hydroxocobalamin (vitamin B12);<br>Biotin;<br>Dietary management.	Stabilized; discharged after 13 days.
**P7**	F	2.5 mo	Vomiting;<br>Hypotonia;<br>Lethargy;<br>Tachypnea; Skin lesion; Poor feeding	13.47 (Elevated)	1.73 (Elevated)	3-HP elevated	184 (Elevated)	204 (Normal)	+	24.7 (Elevated)	94 (Anemia)	268 (Normal)	0.36 (Leukopenia)	107/45 (Elevated)	Mechanical ventilation; Protein restriction;<br>IV glucose infusion;<br>CVVH;<br>Antibiotics;<br>IV L-carnitine;<br>Sodium benzoate; Abanuti;<br>Hydroxocobalamin (vitamin B12); Isoleucine; Valine<br>Biotin;<br>Dietary management.	Stabilized; discharged after 26 days.
**P8**	M	34.0 mo	Vomiting	10.98 (Elevated)	1.61 (Elevated)	PA elevated	164 (Elevated)	536 (Elevated)	−	12.9	103 (Anemia)	32 (Thrombocytopenia)	2.24 (Normal)	35.9/35 (Normal)	Sodium benzoate; Protein restriction;<br>IV glucose infusion;<br>Antibiotics;<br>IV L-carnitine;<br>Hydroxocobalamin (vitamin B12);<br>Biotin;<br>Dietary management.	Stabilized; discharged after 9 days.
**P9**	M	Newborn screening	Hypotonia;<br>Lethargy;<br>Skin lesion; Poor feeding	7.42 (Elevated)	3.68 (Elevated)	3-HP; MCA elevated	134 (Elevated)	N/A	+	16.5 (Elevated)	111 (Normal)	186 (Normal)	3.67 (Normal)	103/67 (Elevated)	Sodium benzoate; Protein restriction;<br>IV glucose infusion;<br>Antibiotics;<br>IV L-carnitine;<br>Hydroxocobalamin (vitamin B12);<br>Biotin; Abanuti;<br>Dietary management.	Stabilized; discharged after 12 days.
**P10**	M	13.0 mo	Vomiting;<br>Hypotonia	N/A	N/A	3-HP; MCA; 3HV;<br>glutaric acid; fumaric acid; HMBA elevated	105 (Elevated)	400 (Normal)	+	17.0 (Elevated)	68 (Severe anemia)	316 (Normal)	3.96 (Normal)	85/47 (Elevated)	Mechanical ventilation; Sodium benzoate; Protein restriction;<br>IV glucose infusion;<br>Antibiotics;<br>IV L-carnitine;<br>Hydroxocobalamin (vitamin B12);	Stabilized; discharged after 24 days.

															Biotin;<br>Dietary management.	
**P11**	F	5.0 mo	Vomiting	17.72<br>(Elevated)	N/A	PG; TG; MCA elevated	93<br>(Elevated)	N/A	-	9.4	129<br>(Normal)	230<br>(Normal)	4.59<br>(Normal)	24/27<br>(Normal)	Mechanical ventilation; Sodium benzoate; Protein restriction;<br>IV glucose infusion;<br>Antibiotics;<br>IV L-carnitine;<br>Hydroxocobalamin (vitamin B12);<br>Biotin;<br>Dietary management.	Stabilized; discharged after 9 days.
**P12**	F	9.0 mo	Vomiting; Lethargy; Seizure	10.13<br>(Elevated)	N/A	PA elevated	73<br>(Elevated)	300<br>(Normal)	+	17.9<br>(Elevated)	123<br>(Normal)	116<br>(Thrombocytopenia)	1.98<br>(Leukopenia)	45/24<br>(Normal)	Mechanical ventilation; Sodium benzoate; Protein restriction;<br>IV glucose infusion;<br>Antibiotics;<br>IV L-carnitine;<br>Hydroxocobalamin (vitamin B12);<br>Biotin; CoQ10<br>Dietary management.	Stabilized; discharged after 15 days.
**P13**	M	0.63 mo<br>(19 days)	Hypotonia; Lethargy; Poor feeding	10.53<br>(Elevated)	2.83<br>(Elevated)	3-HP; MCA; LA;<br>3-HB; AcOH elevated	655<br>(Elevated)	N/A	−	6.9	139<br>(Normal)	8<br>(Thrombocytopenia)	0.74<br>(Leukopenia)	33/26<br>(Normal)	Mechanical ventilation; Sodium benzoate; Protein restriction;<br>IV glucose infusion;<br>CVVH;<br>Antibiotics;<br>IV L-carnitine;<br>Hydroxocobalamin (vitamin B12);<br>Biotin;<br>Antiepileptics (Keppra);<br>Dietary management.	Stabilized; discharged after 33 days.
**P14**	M	0.33 mo<br>(10 days)	Hypotonia;<br>Lethargy;<br>Seizure; Tachypnea;<br>Poor feeding	N/A	N/A	3-HP; HMBA; MCA;<br>AcOH; 3-OH-Val elevated	270<br>(Elevated)	1100<br>(Elevated)	+	18.5<br>(Elevated)	153<br>(Normal)	229<br>(Normal)	2.35<br>(Normal)	31/39<br>(Normal)	Mechanical ventilation; Sodium benzoate; Protein restriction;<br>IV glucose infusion;<br>CVVH;<br>Antibiotics;<br>IV L-carnitine;<br>Hydroxocobalamin (vitamin B12);<br>Biotin;<br>Dietary management.	Stabilized; discharged after 10 days.
**Total**			Lethargy 9/14 (64.3%);<br>Vomiting 8/14 (57.1%);<br>Seizure 6/14 (42.9%); Hypotonia 6/14 (42.9%);<br>Poor feeding 6/14 (42.9%)<br>Tachypnea 5/14 (35.7%);<br>Skin lesion 3/14 (21.4%);	**12/12**<br>(100%)<br>Elevated C3	**10/10**<br>(100%)<br>Elevated C3/C2	**14/14**<br>**(100%)**<br>Abnormal urinary organic acids	**14/14**<br>**(100%)**<br>Hyperammonemia	**4/10**<br>**(40%)**<br>Hyperglycinemia	**9/14**<br>**(64.3%)**<br>Metabolic acidosis	**8/14**<br>**(57.1%)**<br>Elevated	**7/14**<br>**(50%)**<br>Anemia	**3/14**<br>**(21.4%)**<br>Thrombocytopenia	**6/14**<br>**(42.9%)**<br>Leukopenia	**3/14**<br>**(21.4%)**<br>Elevated liver enzymes		

		Hypothermia 1/14 (7.1%)													

Abbreviations: M, male; F, female; +, present; −, absent; Ref, reference range; N/A, not available; NBS, newborn screening; mo, months; Hb, hemoglobin; WBC, white blood cell count; C3, propionylcarnitine; C2, acetylcarnitine; C3/C2, propionylcarnitine-to-acetylcarnitine ratio; 3-HP, 3-hydroxypropionic acid; MCA, methylcitrate; PA, propionic acid; PG, propionylglycine; TG, tiglylglycine; 3HV, 3-hydroxyvaleric acid; HMBA, 2-methyl-3-hydroxybutyric acid; LA, lactic acid; 3-HB, 3-hydroxybutyric acid; AcOH, acetoacetic acid; 3-OH-Val, 3-hydroxyvaleric acid; NH_3, ammonia; AST, aspartate aminotransferase; ALT, alanine aminotransferase; IV, intravenous; CVVH, continuous veno-venous hemofiltration; CoQ10, coenzyme Q10.

Supplementary Table S2. Classification of 10 *PCCB* variants according to recommendations off the American College of Medical Genetics and Genomic (ACMG)

**1. *PCCB*: c.303+3A>T: variant of uncertain significance (PM3_Moderate, PM2_Supporting, PP3, PP4)**		
1 Moderate	PM3_Moderate	The c.303+3A>T variant was observed in one affected proband together with an independently classified pathogenic variant, although the phase was unknown (0.5 points).<br>In an additional affected proband, the variant was identified in the homozygous state, with biparental inheritance confirmed by parental testing (0.5 points).<br>The cumulative PM3 score was therefore 1.0 point, corresponding to PM3_Moderate
3 Supports	PM2_Supporting	Absent (or extremely rare) in 1,000 Genomes Project, Exome Variant Project, Genome Aggregation Database.
	PP3	The c.303+3A>T splice-region variant was predicted to disrupt normal splicing, with a high SpliceAI score of 0.90.
	PP4	Patient's phenotype or family history is highly specific for a disease with a single genetic etiology.
**2. *PCCB*: c.493C>T p.Arg165Trp: Pathogenic (PS3, PM1, PM3_Moderate, PP3_Moderate, PM2_Supporting, PP4)**		
1 Strong	PS3	PMID: 12007220: Chloupkova et al. (2002)[50] showed that Arg165Trp reduced propionyl-CoA carboxylase activity in patient fibroblasts.
3 Moderates	PM1	Arg165 is a highly conserved and functionally vital residue located in the N-terminal region of the PCCB. Gly163, Ala164, and Arg165 are highly conserved catalytic residues located within the carboxyltransferase (CT) domain active site of the PCC.
	PM3_Moderate	The p.(Arg165Trp) variant has been identified in two independent affected probands with propionic acidemia. In an Indian cohort, one patient was homozygous for p.(Arg165Trp) (0.5 points; Gupta et al., 2016, PMID: 27227689)[41]. In a Chinese cohort, another affected patient carried p.(Arg165Trp) together with the pathogenic PCCB variant c.331C>T, p.(Arg111Ter); as the phase was not confirmed, this observation contributed 0.5 points (Liu et al., 2022, PMID: 35331292)[15]. The cumulative PM3 score was therefore 1.0 point, corresponding to PM3_moderate.

	PP3_Moderate	The variant showed a high REVEL score (0.930), supporting moderate computational evidence for pathogenicity; multiple additional algorithms consistently predicted a deleterious effect
2 Supports	PM2_Supporting	Extremely rare in 1,000 Genomes Project, Exome Variant Project, Genome Aggregation Database.
	PP4	Patient's phenotype or family history is highly specific for a disease with a single genetic etiology.
**3. *PCCB*: c.1015A>G p.(Asn339Asp) variant of uncertain significance (PP3_Moderate, PM2_Supporting, PP4)**		
1 Moderate	PP3_Moderate	The p.(Asn339Asp) variant showed a high REVEL score (0.945), supporting moderate computational evidence for pathogenicity; additional in silico tools also predicted a deleterious effect
2 Supports	PM2_Supporting	Absent (or extremely rare) in 1,000 Genomes Project, Exome Variant Project, Genome Aggregation Database.
	PP4	Patient's phenotype or family history is highly specific for a disease with a single genetic etiology.
**4. *PCCB*: c.1087T>C p.(Ser363Pro) Likely Pathogenic (PM3_Strong, PP3_Moderate, PM2_Supporting, PP4)**		
1 Strong	PM3_Strong	PM3_Strong: The p.(Ser363Pro) variant was observed in multiple independent affected individuals with propionic acidemia, including five compound-heterozygous probands carrying independently classified pathogenic or likely pathogenic *PCCB* variants (PMID: 35331292) [15] and one additional homozygous affected fetus with biparental inheritance (PMID: 33028371)[24]. The cumulative PM3 score was therefore 2.75 points, corresponding to PM3_Strong.
1 Moderate	PP3_Moderate	The variant showed a high REVEL score (0.945), supporting moderate computational evidence for pathogenicity; multiple additional algorithms consistently predicted a deleterious effect.
2 Supports	PM2_Supporting	Absent (or extremely rare) in 1,000 Genomes Project, Exome Variant Project, Genome Aggregation Database.
	PP4	Patient's phenotype or family history is highly specific for a disease with a single genetic etiology.
**5. *PCCB*: exon 10 deletion: Pathogenic (PVS1, PM3_Moderate, PM2_Supporting, PP4)**		
1 Very strong	PVS1	The exon 10 deletion causes an out-of-frame alteration, introducing a premature termination codon (PTC) and is expected to result in nonsense-mediated mRNA decay (NMD) and loss of function (LOF); LOF is an established disease mechanism for PCCB

1 Moderate	PM3_Moderate	The exon 10 deletion was confirmed to be in trans with the independently classified PCCB c.1132G>T, p.(Val378Phe) variant in an affected proband by parental testing (1.0 point)
2 Supports	PM2_Supporting	Absent (or extremely rare) in 1,000 Genomes Project, Exome Variant Project, Genome Aggregation Database.
	PP4	Patient's phenotype or family history is highly specific for a disease with a single genetic etiology.
**6. *PCCB*: c.1124C>T p.(Ala375Val): Likely Pathogenic (PM3_Strong, PP3_Moderate, PM2_Supporting, PP4)**		
1 strong	PM3_Strong	The p.(Ala375Val) variant was identified in multiple independent affected probands with propionic acidemia. It was confirmed in trans with an independently classified pathogenic/likely pathogenic PCCB variant in one proband (1.0 point) and with rare variants of uncertain significance in three additional probands (0.25 points each). The variant was also identified in the homozygous state in four affected probands, including two with confirmed biparental inheritance; homozygous observations contributed a maximum of 1.0 point according to the ClinGen SVI recommendations. The cumulative PM3 score was therefore 2.75 points, corresponding to PM3_Strong.
1 Moderate	PP3_Moderate	The p.(Ala375Val) variant showed a high REVEL score (0.972), supporting moderate computational evidence for pathogenicity; multiple additional algorithms consistently predicted a deleterious effect
2 Supports	PM2_Supporting	Absent (or extremely rare) in 1,000 Genomes Project, Exome Variant Project, Genome Aggregation Database.
	PP4	Patient's phenotype or family history is highly specific for a disease with a single genetic etiology.
**7. *PCCB*: c.1132G>T p.(Val378Phe): Likely Pathogenic (PM3_Strong, PP3_Moderate, PM2_Supporting, PP4)**		
1 Strong	PM3_Strong	The p.(Val378Phe) variant was confirmed to be in trans with the independently classified likely pathogenic p.(Ala375Val) variant in two independent affected probands (1.0 point each). In an additional affected proband, p.(Val378Phe) was confirmed to be in trans with a pathogenic exon 10 deletion (1.0 point). The cumulative PM3 score was therefore 3.0 points, corresponding to PM3_Strong
1 Moderate	PP3_Moderate	The p.(Val378Phe)variant showed a high REVEL score (0.977), supporting moderate computational evidence for pathogenicity; multiple additional algorithms consistently predicted a deleterious effect

2 Supports	PM2_Supporting	Absent (or extremely rare) in 1,000 Genomes Project, Exome Variant Project, Genome Aggregation Database.
	PP4	Patient's phenotype or family history is highly specific for a disease with a single genetic etiology.
**8. *PCCB*: c.1232A>G p.(His411Arg): Likely Pathogenic (PM3_Moderate, PP3_Moderate, PM2_Supporting, PP4)**		
2 Moderates	PM3_Moderate	The p.(His411Arg) variant was confirmed to be in trans with the independently classified pathogenic/likely pathogenic p.(Ala375Val) variant in one affected proband by parental testing.
	PP3_Moderate	The p.(His411Arg) variant showed a high REVEL score (0.925), supporting moderate computational evidence for pathogenicity, multiple additional algorithms consistently predicted a deleterious effect.
2 Supports	PM2_Supporting	Absent (or extremely rare) in 1,000 Genomes Project, Exome Variant Project, Genome Aggregation Database.
	PP4	Patient's phenotype or family history is highly specific for a disease with a single genetic etiology.
**9. *PCCB*: c.1534C>T p.Arg512Cys: Pathogenic (PS3, PM3_Strong, PP3_Moderate, PM2_Supporting, PP4)**		
2 Strongs	PS3	Functional studies demonstrated markedly reduced propionyl-CoA carboxylase activity (<10% of normal) and reduced stability of the p.(Arg512Cys) mutant PCCB protein in a PCCB-deficient human fibroblast expression system (PMID: 12757933)[37]
	PM3_Strong	The p.(Arg512Cys) variant has been reported in multiple independent individuals with propionic acidemia, either in the homozygous state or in combination with another disease-causing PCCB variant (PMID: 9683601[14], PMID: 24863100[51], and PMID: 33473339[52]). In the present cohort, an additional affected proband was homozygous for p.(Arg512Cys), with biparental inheritance confirmed by parental testing, contributing an additional 0.5 PM3 points. Overall, the available case-level evidence supports PM3_Strong
1 Moderate	PP3_Moderate	The p.(Arg512Cys) variant showed a high REVEL score (0.921), supporting moderate computational evidence for pathogenicity, multiple additional algorithms consistently predicted a deleterious effect.
2 Supports	PM2_Supporting	Absent (or extremely rare) in 1,000 Genomes Project, Exome Variant Project, Genome Aggregation Database.

	PP4	Patient's phenotype or family history is highly specific for a disease with a single genetic etiology.
**10. *PCCB*: c.1540C>T p.Arg514Ter: Pathogenic (PVS1_Strong, PS3, PM3_Strong, PM2_Supporting, PP4)**		
3 Strongs	PVS1_Strong	The p.(Arg514Ter) variant introduces a premature stop codon near the C-terminus and is predicted to produce a truncated protein. As loss of function is an established disease mechanism for PCCB, PVS1_Strong was applied
	PS3	Experimental studies showed that p.(Arg514Ter) impairs PCCB function, with no full-length protein detected in at least one study (PMID: 11136555)[53]
	PM3_Strong	The p.(Arg514Ter) variant was reported in an affected proband with two *PCCB* variants confirmed in trans (PMID: 27227689; 1.0 point)[41]. An additional affected proband carrying p.(Arg514Ter) together with the independently classified likely pathogenic p.(Ser363Pro) variant, with unknown phase, was reported by Liu et al. (PMID: 35331292; 0.25 points)[15]. In the present cohort, p.(Arg514Ter) was confirmed to be in trans with p.(Ser363Pro) by parental testing (1.0 point). The cumulative PM3 score was 2.25 points, corresponding to PM3_Strong
2 Supports	PM2_Supporting	Absent (or extremely rare) in 1,000 Genomes Project, Exome Variant Project, Genome Aggregation Database.
	PP4	Patient's phenotype or family history is highly specific for a disease with a single genetic etiology.

PP3 strength for missense variants was assigned using REVEL v1.3. REVEL scores of 0.644–0.772 were considered PP3_Supporting, whereas scores ≥0.773 were considered PP3_Moderate. Computational evidence was conservatively capped at Moderate strength.
